# Food budget ratio as an equitable metric for food affordability and insecurity: a population-based cohort study of 121 remote Indigenous communities in Canada

**DOI:** 10.1186/s12889-023-17385-x

**Published:** 2024-01-24

**Authors:** Jennifer Guan, Jeremy C.-H. Wang

**Affiliations:** 1 Department of Family and Community Medicine, Temerty Faculty of Medicine, University of Toronto, 27 King’s College Circle, Toronto, ON M5S 1A1 Canada; 2 Department of Systems Design Engineering, Faculty of Engineering, University of Waterloo, 200 University Ave W, Waterloo, ON N2L 3G1 Canada

**Keywords:** Food insecurity, Food budget ratio, Affordability, Health equity, Public health, Indigenous, Canada

## Abstract

**Background:**

Food insecurity is a public health issue for many regions globally, and especially Indigenous communities. We propose food budget ratio (FBR)—the ratio of food spending to after-tax income—as an affordability metric that better aligns with health equity over traditional price-focused metrics. Existing census and inflation monitoring programs render FBR an accessible tool for future affordability research.

**Methods:**

Public census and food pricing datasets from 2011 to 2021 were analyzed to evaluate food affordability for a cohort of 121 remote Indigenous communities in Canada (*n* = 80,354 persons as of March 2021). Trends in population-weighted versus community-weighted averages, inflation-adjusted mean price of the Revised Northern Food Basket (RNFB), and distributions of FBR, per-capita price of food, and per-capita after-tax income were calculated and compared to Canada at large.

**Results:**

Population-weighted versus community-weighted mean price of the RNFB differed by < 5% for most points in time, peaking at 17%. Mean raw price of the RNFB was relatively stable, while mean inflation-adjusted price of the RNFB decreased 19%. Mean and standard deviation in FBR trended downwards from (0.40; 0.21) in 2011 to (0.25; 0.10) in 2021, while the mean for Canada held stable at 0.10 ± 0.01. Mean and standard deviation in inflation-adjusted per-capita price of food fell from ($5,621; $493) to ($4,510; $243), while the Canada-wide mean rose from $2,189 to $2,567; values for per-capita after-tax income increased from ($17,384; $7,816) to ($21,661; $9,707), while the Canada-wide mean remained between $24,443 and $26,006. Current Nutrition North Canada (NNC) subsidy rates correlate closely with distance to nearest transportation hub (σ_XY_ = 0.68 to 0.70) whereas food pricing, after-tax income, and FBR correlate poorly with distance (σ_XY_ = -0.22 to 0.03).

**Conclusions:**

The FBR approach yields greater insights on food affordability compared to price-based results, while using readily available public datasets. Whereas 19% reductions in RNFB per-capita food price were observed, FBR decreased 63% yet remained 2.5 times the Canada-wide FBR. The reduction in FBR was driven both by the reduced price of food and a 25% increase in after-tax income. It is recommended that NNC consider FBR for performance measurement and setting subsidy rates.

## Introduction

Food insecurity is a longstanding public health issue among Indigenous communities around the world [1, 2]. For Canada’s remote Indigenous communities in particular, studies indicate that roughly half of all remote First Nations and Inuit households experience some level of food insecurity [3, 4]—exact figures vary depending on the region under consideration. This contrasts with the Canada-wide rate of food insecurity of just under 13% [5].

Existing literature focuses heavily on price of food as a key indicator of food affordability. For example, Wendimu et al. [6] compares food pricing in remote communities of Manitoba with that found in the provincial capital, Winnipeg. Galloway [7] discusses various challenges with accurately and consistently measuring food pricing in remote northern Canada. Skinner et al. [8] and Burnett et al. [9] reveal through qualitative semi-structured interviews that those living in remote communities are concerned about the high price of food as well as lower incomes in the north. Moreover, the Government of Canada’s Revised Northern Food Basket (RNFB), established in 2007, has been a popular tool for monitoring the price of food in northern regions. Nutrition North Canada (NNC)—a federal food subsidy program targeting remote Indigenous communities—collects and publishes RNFB data every quarter across the 121 remote communities eligible for NNC subsidies. Many of the studies indicated above consider RNFB data in one form or another, with only some papers that analyzed price in conjunction with other considerations such as availability of particular nutritious foods [6], the amount of government subsidies applied specifically to traditional Indigenous foods [7], or the economics of energy and nutrient density in foods [10]. Meanwhile, other studies have reported on the causes and qualitative aspects of food insecurity, providing context on the complex socioeconomic structures therein [9].

However, the price of food alone is a limited metric for food insecurity. Some studies have not accounted for inflation over long time horizons [7] or when considering historical price data in a contemporary time period [3], while government reporting based on statistical data has not always adjusted prices for inflation [11]. Foods of the same price can also yield very different rates of food insecurity between two households in distinct communities, or even within the same community, due to differences in after-tax household income. As well, opportunities to participate in wage-based economies may vary significantly from one community to the next, resulting in different distributions of household incomes between different communities. It is especially common to observe lower household incomes and higher food prices in remote regions. Indeed, the principle of equality of expenditure has long been criticized in the literature for clashing with equality of access in healthcare [12]. While more complex models and measures for food insecurity have been proposed over the years [13–15], these have generally not been incorporated into NNC’s policy planning and program evaluation practices.

There is therefore a need to investigate new food affordability indicators that contextualize food pricing in terms of health equity, and which can be easily measured and integrated into government policy and programs. Braveman [16] defines health equity as “the principle underlying a commitment to reduce—and, ultimately, eliminate—disparities in health and its determinants, including social determinants”. Under this definition, current literature provides an incomplete picture of remote Indigenous health equity since price is only one of many social determinants of nutritional health. Other factors include household income, access to transportation, access to traditional hunting or harvesting, and awareness of food subsidy programs, among other possible considerations [17].

In this study, we propose food budget ratio (FBR) as a food affordability metric that more closely aligns with health equity compared to traditional price-only indicators. We define FBR as the ratio of food spending to after-tax income, thus representing the buying power that households allocate toward food. Compared to food prices, which do not necessarily correlate with a household’s capacity to purchase food, FBR assesses food expenditure against economic freedom. By also accounting for inflation and leveraging improved averaging techniques, we hope that FBR will serve as a more equitable metric for future studies on food affordability.

To demonstrate the usefulness of FBR and update the current knowledge base on remote Indigenous food insecurity, we use FBR in this study to investigate the affordability of nutritious foods across Canada’s remote Indigenous communities. The results lend themselves to various recommendations with respect to policy and program evaluation of the NNC food subsidy program.

## Methods

### Overview of study design

This study was designed to leverage existing publicly available datasets and glean new insights by adopting the health equity lens furnished by an FBR analysis. First, we researched publicly available datasets with relevant food pricing, inflation, and household information. We then scrutinized these datasets for completeness and transparency. Since these data are normally published on a per-community basis as a matter of simplicity and to protect the privacy of the relatively small communities under consideration, population-weighted as well as community-weighted averages were calculated, compared, and discussed. Then, raw and inflation-adjusted food pricing trends were computed and analyzed. Finally, distributions of FBR and its underlying variables were analyzed at three intervals coinciding with Canada’s national census (2011, 2016, 2021). The ensuing results and discussion are provided later in this paper.

### Data collection, variables, and sampling

Data from publicly available sources were downloaded, spanning the period March 2011 to March 2021. The sources of data are summarized in Table 1, along with variables of interest and the times when data was measured.

**Table 1:** Sources of publicly available data used in this study along with variables of interest

Name	Publisher	Variables of Interest	Time Period
Census of Population 2011 [18]	Statistics Canada	Population size per census subdivision, median household size	Data collected May 2011, published in 2012
National Household Survey 2011 [19]	Statistics Canada	Median after-tax household income	Data collected May 2011, published in 2012
Census of Population 2016 [20]	Statistics Canada	Median after-tax household income, median household size, population size per census subdivision	Data collected May 2016, published in 2017
Census of Population 2021 [21]	Statistics Canada	Median after-tax household income, median household size, population size per census subdivision	Data collected May 2021, published in 2022
Seasonally adjusted Consumer Price Index (CPI) [22]	Statistics Canada	CPI for all items, CPI for food (reference year 2002 has an index of 100)	Data collected March 2011 to March 2021, sampled and published monthly
Survey of Household Spending [23]	Statistics Canada	National average expenditure per household for food purchased from stores (i.e. groceries)	Data collected 2011 to 2019, sampled annually, published biennially
Cost of the Revised Northern Food Basket (RNFB) [24]	Nutrition North Canada	Price of the RNFB across 121 remote Indigenous communities	March 2011 to March 2021, sampled and published quarterly

The primary reasons that we selected these datasets were: public availability, transparent data management protocols, and the availability of similar datasets in other countries where comparable studies may be conducted in the future. Statistics Canada publishes the data collection and coding process, including considerations for privacy and explanations of gaps in data, with each census. Statistics Canada’s census program collects data every 5 years, analogous to the census programs operated by the U.S. Census Bureau [25] and the Australian Bureau of Statistics [26], which occur every ten and 5 years respectively. Additionally, the NNC program regularly monitors the price of the RNFB as part of its program evaluation efforts. The price data is provided by retailers registered with NNC who are subject to randomized audits to ensure subsidies are passed onto consumers in a compliant fashion. The exact names of retailers contributing data, substitution methodologies for missing data or food basket items in certain communities, and explanation of gaps in data are also published with each dataset [24]. It is important to note that the NNC program came into effect in March 2011, and thus detailed food pricing data is not available before this date. Also, the most recent Canadian census was performed in 2021, hence the 2011 to 2021 time period considered in this study. From March 2011 to March 2021, the total population across all 121 communities studied increased from *n* = 70,374 to *n* = 80,354.

Due to the different sampling frequencies of the datasets, we applied interpolation and downsampling accordingly to analyze data on a quarterly basis aligned with the timing of the RNFB price data. The quarterly reporting frequency was selected to balance the need for granularity and ease of interpretation with respect to data visualization on graphs and discussing macroscopic trends. The Census of Population data and Survey of Household Spending data were linearly interpolated between available data points to estimate quarterly values. The CPI data was downsampled from monthly to quarterly to align with the time basis of the RNFB data. We selected seasonally adjusted—rather than unadjusted—CPI data in order to isolate long-term trend cycles from localized biases and errors in time series pricing data [27].

Of the 121 remote communities for which RNFB data is available between March 2011 and March 2021, 93 communities were registered with the program at inception in March 2011 (78 were eligible for the full subsidy, 15 were eligible for partial subsidies). The remaining 28 communities were onboarded throughout the life of the program, with the majority of these added in October 2016. Five communities were also permanently removed from the program between approximately October 2016 and June 2018 for unknown reasons. Since the resulting time gaps span multiple years and are not fully bounded by known data, we chose not to interpolate or extrapolate due to the inherent uncertainty in reconstructing such data for such extended time periods. Instead, missing data points were omitted from further calculations.

In addition, the 2011, 2016, and 2021 census datasets contained occasional omissions and redactions. Some communities were not surveyed as local governments did not grant permission for the survey to be conducted, or surveys were interrupted prior to completion. Forest fires—which tend to span late spring to early fall in Canada—prevented or interrupted Statistics Canada staff from surveying certain communities. Due to the small population size of some communities, data on household income and/or household size was redacted by Statistics Canada to protect the privacy and confidentiality of community members. Wherever possible, we interpolated between measured data points. However, data omissions in 2011 and/or 2021 were not extrapolated due to the lack of bounding data, similar to the principle applied earlier to any missing RNFB data. Census data from 2011 and/or 2021 were thus excluded from any related calculations.

### Population-weighted average

The population-weighted average price of a notional food basket (e.g. the RNFB) may be computed as:

$$\overline{P }=\frac{1}{M}\sum\limits_{i=1}^{N}{P}_{i}{m}_{i}$$

where $\overline{P }$ is the population-weighted average of the food basket, ${P}_{i}$ and ${m}_{i}$ are the food basket price and population size of the ${i}^{th}$ community, $M$ is the total population size across all communities, and $N$ is the total number of communities. The population-weighted average may be computed for each geographic region that is of interest, i.e. a particular province, state, territory, or overall country.

In this study, we compared population-weighted price of the RNFB against NNC’s reported average price of the RNFB—the latter instead weighs each community equally and does not account for the different population sizes that can range from tens of residents to over 1000 residents. The average price of the RNFB within each province and across all communities in Canada was analyzed from 2011 to 2021. Overall trends and differences between the population-weighted versus community-weighted averages are discussed later in this study.

### Inflation-adjusted pricing and household income

The population-weighted food pricing data of the prior step can further be adjusted for inflation according to seasonally adjusted CPI charts. Whereas raw prices offer insight on how the price of food is changing from the consumer’s day-to-day perspective, the inflation-adjusted values permit pricing data to be interpreted with respect to the changing purchasing power of the dollar over time.

For the purposes of the present study, inflation adjustments to the price of the RNFB—as well as to household income, which is required for computing FBR—were calculated as:

$${P}_{2011}=\frac{CP{I}_{2011}}{CP{I}_{x}}{P}_{x}$$

where ${P}_{2011}$ is the dollar amount expressed in 2011 Canadian dollars, $CP{I}_{2011}$ is the value of the CPI index for the relevant commodity group (e.g. CPI for food, or CPI across all items) in 2011, $CP{I}_{x}$ is the value of the CPI index for the relevant commodity group in the year $x$, and ${P}_{x}$ is the dollar amount in year $x$. Thus, in this study, the remaining economic analyses are conducted using 2011 Canadian dollars.

### Food budget ratio

With inflation-adjusted pricing data available, it is possible to define a household’s annual food budget ratio (FBR):

$$FB{R}_{i}=\frac{{P}_{i}\times N}{{H}_{i}}\times \frac{{S}_{i}}{{S}_{FB}}$$

where $FB{R}_{i}$ is the food budget ratio for the ${i}^{th}$ community of interest, ${P}_{i}$ is the price of a notional food basket $FB$, $N$ is the number of times a household would theoretically purchase the notional food basket $FB$ over the course of a year (e.g. 52 times if each food basket is assumed to last 1 week), ${H}_{i}$ is the median after-tax annual household income, ${S}_{i}$ is the average household size, and the ${S}_{FB}$ is the assumed household size associated with the notional food basket $FB$. The factor ${S}_{i}/{S}_{FB}$ thus scales the annual household food expenditure ${P}_{i}\times N$ based on the ratio of household sizes assumed in ${P}_{i}$ versus ${H}_{i}$. Inflation-adjusted price of food and household income should be used—in this sense, FBR is also less susceptible to measurement errors associated with the inflation index itself as both the numerator and denominator are scaled by the same factor.

FBR therefore represents the fraction of household purchasing power that is hypothetically required to purchase a notional food basket for the average-sized household of the community. An FBR of zero indicates minimal purchasing power is allocated toward food, either because food is relatively inexpensive or after-tax income is relatively high in that community. Meanwhile, a high FBR would indicate a high price of food relative to the after-tax income of the average household in that community. FBR is therefore an equitable metric as compared to the purely economic metric of food pricing in the absence of after-tax household income. FBR also better measures affordability compared to monitoring food pricing alone.

Additionally, it is possible to calculate and interpret FBR on an annual per-capita basis:

$$FB{R}_{i}=\frac{{\widehat{P}}_{i}}{{\widehat{H}}_{i}}$$

Where ${\widehat{P}}_{i}=\frac{{P}_{i} \times N}{{S}_{FB}}$ is the price of food per household member per year, and ${\widehat{H}}_{i}=\frac{{H}_{i}}{{S}_{i}}$ is the median after-tax annual income per household member. This simplified form is useful to analyze variations in FBR with respect to the underlying variations in per-capita price of food and after-tax income on an annual basis. Household size appears in the earlier calculation of FBR only because food baskets are often designed around households, not individuals.

For this paper, the distribution of FBR across all 121 NNC-eligible communities was analyzed in 2011, 2016, and 2021, aligned with the availability of raw census data. In the case of the RNFB, the assumed household size ${S}_{FB}$ is four individuals. The distribution of FBR was analyzed in terms of mean and standard deviation at each snapshot in time, as well as discussed in terms of longitudinal trends in the inflation-adjusted price of food as measured by FBR.

The longitudinal evolution of FBR is further analyzed with respect to changes in price of food and after-tax income, thus attributing any improvement or worsening of food equity to underlying effects.

## Results

**Fig. 1:**
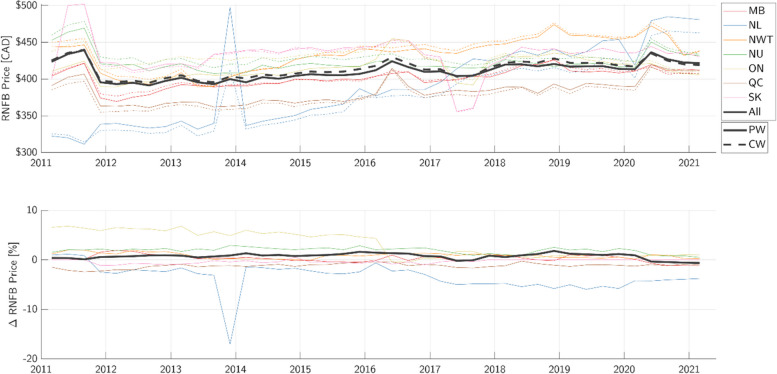
Comparison of average price of the Revised Northern Food Basket (RNFB), grouped by province and across all communities. Top: community-weighted mean price of the RNFB as used by Nutrition North Canada (NNC) (dotted lines) versus the population-weighted mean of the RNFB (solid lines) proposed in this study. Bottom: the percentage difference between community-weighted and population-weighted averages, normalized by the population-weighted average. MB = Manitoba, NL = Newfoundland and Labrador, NWT = Northwest Territories, NU = Nunavut, ON = Ontario, QC = Quebec, SK = Saskatchewan, PW = Population-Weighted, CW = Community-Weighted

The population-weighted mean price of the RNFB, grouped by region and computed nationally across all remote communities, is shown in Fig. 1. The population-weighted mean is plotted against the NNC mean for comparison. The percentage difference between the population-weighted and NNC averages is also shown. Population-weighted versus community-weighted mean price of the RNFB differed by up to 5% for most points in time, peaking at 17%.

**Fig. 2:**
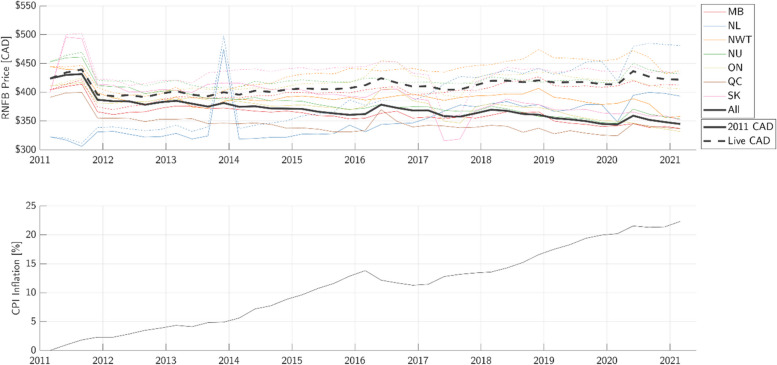
Comparison of raw versus inflation-adjusted population-weighted mean price of the Revised Northern Food Basket (RNFB). Top: the population-weighted mean price of the RNFB, with solid lines indicating the inflation-adjusted values and dotted lines showing live (i.e. raw) values. Bottom: graph of inflation with respect to 2011 as the base year, determined using the food commodity group under the Consumer Price Index (CPI)

The inflation-adjusted population-weighted mean price of the RNFB, grouped by region and computed nationally, is shown in Fig. 2. The inflation-adjusted data is plotted against the raw data for comparison. Also shown is a graph of inflation in the food category of the CPI relative to 2011 as the base year. Mean raw price of the RNFB was relatively stable, while mean inflation-adjusted price of the RNFB fell by 19%.

**Fig. 3:**
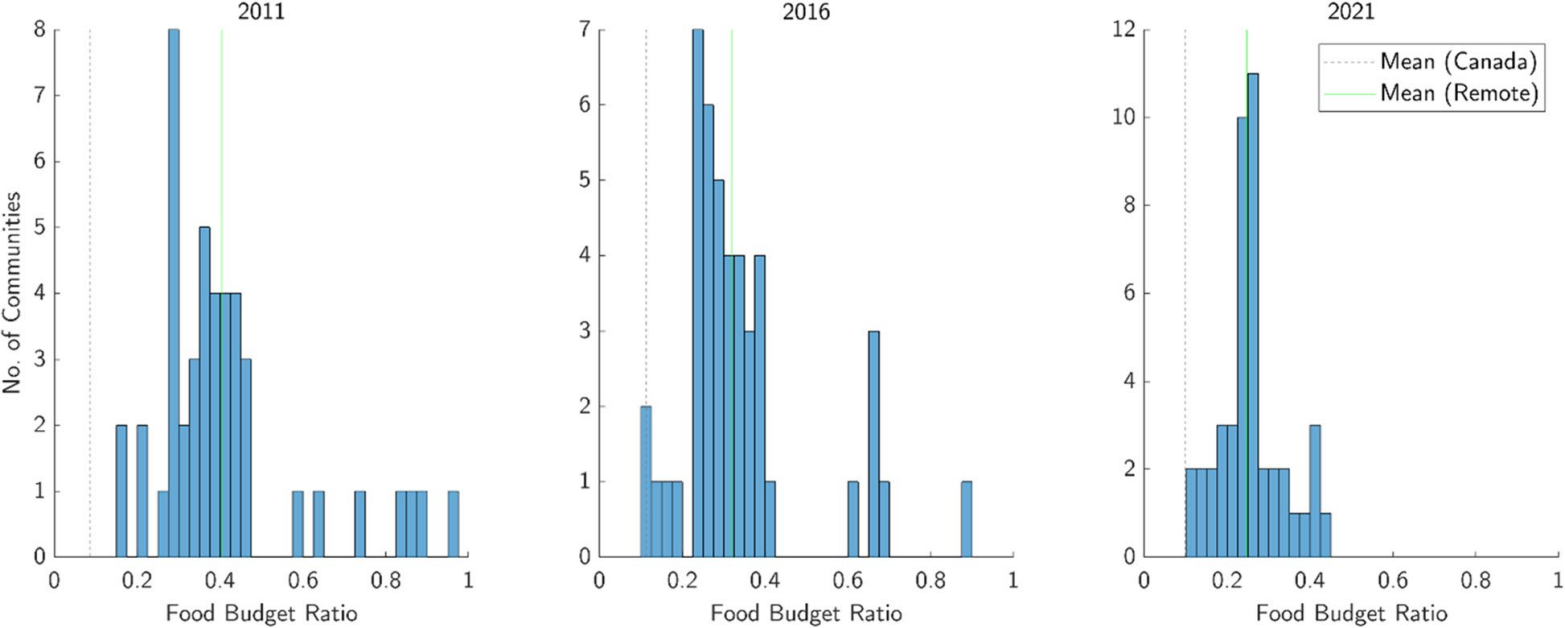
Distribution of Food Budget Ratio (FBR) across all remote communities in 2011, 2016, and 2021. For comparison, the population-weighted mean FBR for remote communities is shown with the mean FBR for the entire population of Canada

**Fig. 4:**
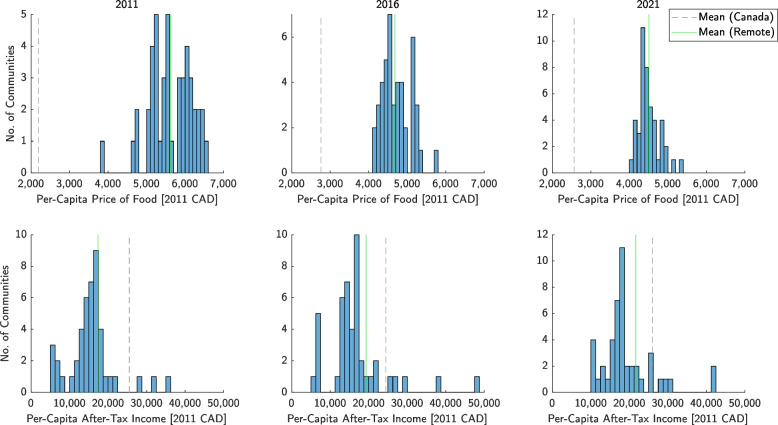
Distribution of inflation-adjusted annual per-capita price of food and annual after-tax income across all remote communities (expressed in 2011 CAD) in 2011, 2016, and 2021. For comparison, the population-weighted mean values for remote communities are shown with the mean values for the entire population of Canada

The distribution of FBR across all 121 NNC-eligible remote Indigenous communities is plotted in Fig. 3, comparing the state of food affordability in 2011, 2016, and 2021. Appropriate inflation-adjustment against CPI for food and the overall CPI was performed on food prices and household income, respectively. Additionally, Fig. 4 shows the distribution in inflation-adjusted annual per-capita price of food and after-tax income, which help to determine FBR. A summary of the means and standard deviations in FBR, per-capita price of food, and per-capita after-tax income are compiled in Table 2, with Canada-wide values provided for reference.

**Table 2:** Mean $\mu$ and standard deviation $\sigma$ of inflation-adjusted annual Food Budget Ratio (FBR), per-capita price of food (in 2011 CAD), and per-capita after-tax income (in 2011 CAD) across all communities in 2011, 2016, and 2021. For reference, the mean FBR of the entire population of Canada is included

Variable	2011		2016		2021	
	$$\mu$$	$$\sigma$$	$$\mu$$	$$\sigma$$	$$\mu$$	$$\sigma$$
FBR for remote communities	0.40	0.21	0.32	0.18	0.25	0.10
FBR for Canada	0.09	-	0.11	-	0.10	-
Per-capita price of food for remote communities	$5,621	$493	$4,679	$322	$4,510	$243
Per-capita price of food for Canada	$2,189	-	$2,759	-	$2,567	-
Per-capita after-tax income for remote communities	$17,384	$7,816	$19,326	$10,082	$21,661	$9,707
Per-capita after-tax income for Canada	$25,517	-	$24,443	-	$26,006	-

Mean and standard deviation in FBR trended downwards from (0.40; 0.21) in 2011 to (0.25; 0.10) in 2021, while the mean for Canada was stable at 0.10 ± 0.01. Mean and standard deviation in inflation-adjusted per-capita price of food fell from ($5,621; $493) to ($4,510; $243), while the mean for Canada rose from $2,189 to $2,567; values for per-capita after-tax income increased from ($17,384; $7,816) to ($21,661; $9,707), while the Canada-wide mean remained between $24,443 and $26,006.

**Fig. 5:**
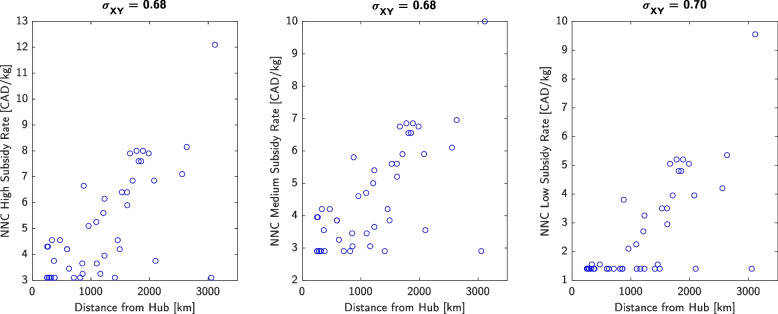
NNC’s high, medium, and low subsidy rates versus distance of each community to the nearest major transportation hub (expressed in 2011 CAD) as of 2021. The correlation coefficients are shown at the top of each plot, indicating strong correlation between subsidy rates and geographical remoteness in terms of distance

**Fig. 6:**
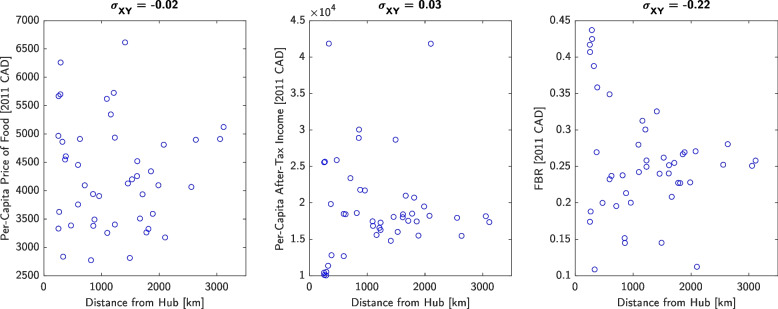
Per-capita price of food, per-capita after-tax income, and FBR in 2021 versus distance of each community to the nearest major transportation hub (expressed in 2011 CAD) as of 2021. The correlation coefficients are shown at the top of each plot, indicating low or no correlation between price, income, or FBR and geographical remoteness in terms of distance

Finally, Fig. 5 plots current NNC subsidy rates versus distance of each community to the nearest transportation hub, while Fig. 6 plots food price, after-tax income, and FBR versus distance. Current subsidy rates correlate well with distance despite the fact that price, income, and FBR do not correlate with distance.

## Discussion

### Community-weighted versus population-weighted averaging

Figure 1 shows that the difference in average price of the RNFB across all communities is generally less than 1% between the community-weighted versus population-weighted mean. However, the averages for individual regions differ as much as 5% in most years. The community-weighted average peaks at 17% less than the population-weighted average, as observed in late 2013 for remote communities in Newfoundland. Thus, it is evident that the choice of averaging technique has a non-negligible impact on the interpretation food pricing by region.

For the purposes of measuring health equity, we therefore recommend that NNC and future studies use population-weighted averaging in the future. A population-weighted average weighs each person equally as opposed to weighing each community equally, and would therefore be more consistent with implication of individual equality found in Canadian constitutional law [28]. Equally weighing two communities of different size can skew the interpretation of food pricing, resulting in a skewed interpretation of food pricing for the average person. However, if one community were to grow significantly in population compared to the rest, this could warrant an important conversation regarding whether and to what extent more populous communities ought to receive greater support from the Canadian government.

###  Raw versus inflation-adjusted price of food

Figure 2 reveals that, on an inflation-adjusted basis, the population-weighted mean price of the RNFB across all communities decreased by 19% from 2011 to 2021. Accordingly, the pricing trends appear different when analyzing raw versus inflation-adjusted data. For instance, the raw (i.e. live) price of the RNFB increased from 2011 to 2021 across Quebec remote communities, whereas the inflation-adjusted data decreased over the same period. This suggests that while the raw price of food in most regions has remained relatively stable or grown slightly, the effective price of food has decreased since 2011. Considering that net inflation during this time was just over 22%, this 19% decrease in the inflation-adjusted average price of food may be attributed at least in part to the NNC subsidy.

When interpreting these results, it must be noted that Statistics Canada’s CPI is based on Canada-wide inflation, and 82% of Canada’s population lives in cities [29]. On the other hand, remote northern retailing often behaves differently from urban retailing owing to the unique transportation, warehousing, and other supply chain considerations [30]. It is thus not possible with the available data to quantify exactly how much of the observed decrease in the inflation-adjusted price of the RNFB may be attributed to NNC subsidies versus other economic factors specific to remote communities.

We therefore recommend that NNC also publish the pre-subsidy price of the RNFB. This would provide a consistent basis for interpreting pre- and post-subsidy pricing and for accurately quantifying the impact of NNC subsidies. NNC does publish actual subsidies issued to each community on a quarterly basis, however actual consumer purchasing behaviour likely varies from the assumptions of the RNFB.

Finally, it is worth noting that the official mandate of the NNC program is “to help make perishable, nutritious food more accessible and more affordable than it otherwise would be to residents of eligible isolated northern communities without year-round surface (road, rail, or marine) access” [31]. The NNC performance measurement strategy framework [32] lists the annual trend and annual average value of the RNFB as key performance indicators, whose targets are “at or below the annual trend… for the [CPI] basket for food” and “at or below the Program launch baseline (2010–2011)” of $438. In this sense, the key performance targets have been satisfied during this timeframe based on the reductions to the inflation-adjusted price of food. However, as we shall observe in the next subsection, trends in food price behave differently from trends in food affordability.

### Food budget ratio, per-capita price of food, and per-capita after-tax income

As noted earlier, FBR tracks affordability by weighing price of food against after-tax household income. Although the previous two subsections indicate that raw RNFB prices were relatively stable and inflation-adjusted prices have fallen since 2011, it is evident from Fig. 3 that the mean FBR for remote communities (0.25) remains more than double the FBR for Canada at large (0.10) as of 2021. Still, the mean FBR for remote communities fell from 0.40 to 0.25 from 2011 to 2021, and the standard deviation of FBR across all communities fell from 0.21 to 0.10. Meanwhile, the FBR for Canada at large remained relatively stable between 0.09 to 0.11. This improvement in FBR for remote communities is arguably more relevant to affordability and a more significant observation than the earlier noted 19% reduction in mean inflation-adjusted price of the RNFB.

Figure 4 shows that while the mean inflation-adjusted per-capita price of the RNFB also fell by 19% from 2011 to 2021, the mean inflation-adjusted per-capita after-tax income simultaneously rose by 25% over the same time period. At a minimum, this suggests that the reduction of FBR in remote communities is not only due to the fall in effective price of food, but also a growth in individuals’ and households’ buying power. Changes in income have clearly had an influential role in affordability trends, arguably more so than the price of food itself during this period specifically. By contrast, the mean price of food for Canada at large actually increased by 17% from 2011 to 2021, while the after-tax income held relatively steady with a moderate 2% growth.

We recommend that NNC consider FBR as a metric of affordability of healthy perishable foods. For example, by monitoring the median, maximum, minimum, and standard deviation of FBR across all remote Indigenous communities, NNC could more closely monitor and mitigate challenges with food affordability. Possible targets could then be benchmarked off Canada-wide values in consultation with community stakeholders. Moreover, the data collected for these new performance metrics would feed directly back into subsidy design.

We also recommend that NNC consider setting each community’s subsidy rates based on the community’s FBR and not geographical latitude or degree of remoteness. The current NNC subsidy rates show a close correlation with distance to the nearest major transportation hub (Fig. 5), despite the absence of correlation between food price, income, or FBR with respect to distance (Fig. 6). For example, Norman Wells is 674 km from the hub of Yellowknife and the average household had 2.5 members and earned an after-tax household income of $122,000 in 2021—whereas the Cat Lake First Nation is 178km from the hub of Sioux Lookout and the average household had 4.6 members with an after-tax income of $62,000. Yet, subsidy rates for Norman Wells are $1.00 to $3.45/kg, while rates for Cat Lake are $1.00 to $2.90/kg according to the NNC website [33]. Remoteness affects each community differently, with affordability driven by factors beyond geography.

Finally, it is worth noting that CIRNAC, the lead agency responsible for NNC, spent only about 80% of its annual budgeted expenditures on NNC in the 2021–2022 fiscal year [34], leaving approximately $30M in unspent funds which could be allocated to additional subsidies for new or existing communities. Although the exact reasons for historical underspending are not publicly disclosed, the full utilization of NNC’s planned budget could increase its annual retail subsidy budget by as much as 23%. The two other funders and partners of NNC—namely Indigenous Services Canada (ISC) and the Public Health Agency of Canada (PHAC)—have also historically spent only about 90% of their planned budgets on nutritional awareness and education initiatives [34]. The full use of their funds could further supplement the proposed subsidy and program metric revitalization by supporting community consultations, stakeholder engagement, and associated awareness efforts.

### Limitations of the study

Several limitations must be noted with this study. First and foremost, the RNFB is a hypothetical food basket that permits uniform comparison between households and communities at the expense of some realism—true purchasing behaviour is likely to be different and quite varied. Without further data on actual dietary choices, it is unknown whether affordability as measured using the RNFB aligns with affordability as experienced by community members.

Secondly, the pricing data used in this study was collected by NNC in collaboration with NNC suppliers who furnish this data on a quarterly basis. This pricing information is based on in-community brick-and-mortar retail stores and does not account for food that is ordered direct-to-consumer—approximately 80% of food is bought in-store while 20% is ordered direct [35]. It is also unknown if this pricing data is an average of each quarter or taken at one point in time, or whether it accounts for weekly discounts or sales that are often promoted in store flyers.

Thirdly, this study focused primarily on the means and standard deviations of FBR and related metrics across communities, due to limited data on distributions within each community’s population. Subject to data availability, future researchers may wish to also consider what fraction of a community’s population meets some criteria related to food affordability (e.g. percentage of population whose FBR is above some threshold).

Fourthly, the extent to which incomplete census data may have impacted results and interpretation cannot be quantified, however the reasons for incomplete data were disclosed by Statistics Canada and are acknowledged at the end of §2.2.

Also notable is that the global COVID-19 pandemic overlapped with the 2021 Census of Population, and NNC also received an additional $25M in funding to further support remote communities [35]. The pandemic’s disruption of supply chains, coupled with additional stimulus and recovery funding, warrants caution when interpreting data from 2020 onward. It is unknown to what extent stimulus and recovery funding may have been provided to those living in remote regions, through programs such as the Canada Emergency Response Benefit and the On-Reserve Income Assistance Program. Nevertheless, the qualitative trends observed from 2011 to 2021 were also observed for the 2011 to 2016 timeframe albeit with different numerical values.

### Future directions

The key policy recommendations for CIRNAC and NNC from this paper may be summarized as:

1. Use population-weighted averaging—either in addition or instead of—the current community-weighted averaging, which would help weigh the needs of each person equally.
2. Publish both the pre- and post-subsidy price of the RNFB in order to help distinguish the impact of subsidies on food affordability at each point in time in addition to longitudinally.
3. Update the mandate of NNC to focus on bringing remote Indigenous food affordability further in line with national averages, rather than the current mandate which is focused on inflationary mitigation.
4. Update the metrics of NNC to include FBR as a more equitable metric for food affordability. The data to monitor FBR is already collected through existing government programs.
5. Set subsidy rates with the help of food affordability metrics such as FBR, and reduce the importance of geographical remoteness—neither price, income, nor FBR correlate with remoteness.
6. Utilize the full budget available to NNC to ensure the greatest quantity of subsidies can be passed onto those living in remote Indigenous communities.

It must be emphasized that no one metric can fully capture the complexity of food insecurity [36]. The goal of this research was to advance the equity of food affordability, as demonstrated by studying FBR among a cohort of 121 remote Indigenous communities in Canada. However, the availability, cultural relevance, logistics, and freshness of nutritious foods, as well as awareness of food security programs, are just some of the additional considerations that are critical but beyond the scope of this study. Future studies should continue to investigate these multifaceted factors and map their independent and joint effects on food security.

The FBR approach can also continue to be explored for studying food affordability in other populations—such as Canada or other countries at large, or at-risk subpopulations and regions (e.g. low-income, remote, Indigenous, experiencing conflict). The data sources acknowledged in §2.2 could just as well be leveraged to monitor FBR for other segments of the Canadian population, provided that the price of an appropriate food basket is available instead of the RNFB. We hope that the present contributions will encourage researchers, policymakers, and the public to adopt equitable metrics when addressing the social determinants of health.

## Data Availability

All data generated or analysed during this study are included in this published article and its supplementary information files.
